# Regulation of NKG2D-Dependent NK Cell Functions: The Yin and the Yang of Receptor Endocytosis

**DOI:** 10.3390/ijms18081677

**Published:** 2017-08-02

**Authors:** Rosa Molfetta, Linda Quatrini, Angela Santoni, Rossella Paolini

**Affiliations:** 1 Department of Molecular Medicine, Sapienza University of Rome, Laboratory Affiliated to Istituto Pasteur Italia—Fondazione Cenci Bolognetti, Viale Regina Elena 291, 00161 Rome, Italy; rosa.molfetta@uniroma1.it (R.M.); quatrini@ciml.univ-mrs.fr (L.Q.); angela.santoni@uniroma1.it (A.S.); 2 Centre d’Immunologie de Marseille-Luminy, Aix Marseille Université UM2, Inserm, U1104, CNRS UMR7280, 13288 Marseille, France

**Keywords:** innate immune system, natural killer (NK) cells, NK cell receptors

## Abstract

Natural-killer receptor group 2, member D (NKG2D) is a well characterized natural killer (NK) cell activating receptor that recognizes several ligands poorly expressed on healthy cells but up-regulated upon stressing stimuli in the context of cancer or viral infection. Although NKG2D ligands represent danger signals that render target cells more susceptible to NK cell lysis, accumulating evidence demonstrates that persistent exposure to ligand-expressing cells causes the decrease of NKG2D surface expression leading to a functional impairment of NKG2D-dependent NK cell functions. Upon ligand binding, NKG2D is internalized from the plasma membrane and sorted to lysosomes for degradation. However, receptor endocytosis is not only a mechanism of receptor clearance from the cell surface, but is also required for the proper activation of signalling events leading to the functional program of NK cells. This review is aimed at providing a summary of current literature relevant to the molecular mechanisms leading to NKG2D down-modulation with particular emphasis given to the role of NKG2D endocytosis in both receptor degradation and signal propagation. Examples of chronic ligand-induced down-regulation of NK cell activating receptors other than NKG2D, including natural cytotoxicity receptors (NCRs), DNAX accessory molecule-1 (DNAM1) and CD16, will be also discussed.

## 1. NKG2D/NKG2D Ligand Axis in the Recognition of Damaged Cells

Activation of natural killer (NK) cells is dictated by a balance between negative signals provided by inhibitory receptors upon interaction with major histocompatibility complex (MHC) class I molecules and positive signals promoted by a variety of activating receptors [1]. Besides the low affinity receptor for IgG (FcγRIIIA or CD16), that is responsible for the antibody-dependent cell cytotoxicity (ADCC), NK cells express a wide array of activating receptors that cooperate in driving the natural cytotoxic response. These receptors include the natural cytotoxicity receptors (NCRs), the signaling lymphocyte activation molecule (SLAM) family receptor member, 2B4, the Ig-like receptor DNAX accessory molecule-1 (DNAM1), and the lectin-like receptor natural-killer receptor group 2, member D (NKG2D).

NKG2D is a potent activating receptor constitutively expressed on all NK cells but is also present on invariant natural killer T (NKT) cells and subsets of T cells including CD8^+^ αβ T cells, and γδ T cells. It can bind several ligands poorly expressed on healthy cells, but is up-regulated upon stressing stimuli in the context of cancer or viral infection [2,3,4,5]. Several in vivo models support a fundamental role for the NKG2D receptor in NK cell responses toward abnormal cells. Ectopic expression of NKG2D ligands (NKG2DLs) causes tumor rejection in syngeneic mouse models despite MHC class I expression [6,7], while NKG2D deficiency impairs surveillance towards spontaneous malignancies [8,9].

NKG2D functions as co-stimulatory rather than a stimulatory receptor: on human CD8^+^ T cells it potentiates T cell receptor (TCR)-induced cytotoxic function [10], while on freshly isolated human NK cells its engagement alone is not sufficient to promote full NK cell functional responses [11]. In particular, the synergistic co-engagement of 2B4 receptor is required to overcome negative signals mediated by inhibitory receptors [12,13]. To propagate signals, human NKG2D needs to associate with the transmembrane adaptor DNAX activating protein 10 (DAP10) [14], which contains a tyrosine-based motif (YINM). Ligand binding promotes the phosphorylation of YINM domain allowing the recruitment of the growth factor receptor-bound protein 2 (Grb2)/Vav1 complex and the activation of phosphatidyl-inositol-3-kinase (PI3K) [14,15]. PI3K promotes survival pathways through the activation of Akt, while the recruitment of Grb2/Vav1 complex allows phosphorylation of Vav1 and the consequent activation of Phospholipase C gamma (PLCγ2), and of the Src homology 2 (SH2) domain-containing leukocyte protein of 76 kD (SLP-76) [16].

Upon activation, murine NK cells express an alternatively spliced isoform (NKG2D-S) that can associate with DAP10 or DAP12 adaptor proteins. The presence of an immune tyrosine-based activation motif (ITAM) in DAP12 cytoplasmic region allows the recruitment of Syk and ZAP70 tyrosine kinases and the consequent PLCγ phosphorylation and PI3K activation [17,18].

The most remarkable characteristic of NKG2D receptor resides in its ability to bind to a large repertoire of self-proteins induced by stress pathways, thus mediating the “induced self” recognition [2,3,4,5]. In humans, these ligands include the highly polymorphic MHC class I related proteins (MIC)A and MICB, and 6 members of UL16 binding proteins (ULBP). In mice only genes orthologous to the human *ULPB/RAET*1 family are present, and encode three subgroups of proteins: the GPI-linked RAE-1 (retinoic acid early inducible-1), MULT1 (murine UL16-binding protein-like transcript 1) and H60 ligands [3,4].

NKG2DLs are absent on the surface of the vast majority of healthy tissues but are up-regulated under stressing conditions, including mitosis, viral infection and cancer by several pathways mainly acting at transcriptional and post-transcriptional levels [3,19,20].

The activation of the DNA Damage Response (DDR) is recognized as the major signalling pathway responsible for the transcriptional up-regulation of NKG2DL expression [3,19,20,21,22]. This pathway, initiated by ataxia telangiectasia mutated (ATM), ATM and Rad3-related (ATR) and the DNA-dependent protein kinase (DNA-PK), promotes NKG2DL expression on healthy proliferating cells [23,24]. Moreover, DDR-mediated pathway may be activated upon infection by several viruses, including herpes viruses, adenoviruses and retroviruses, and is responsible for NKG2DL up-regulation. However, while a direct link between these two phenomena has been established for HIV-1 [25,26], up-regulation of MICA upon human cytomegalovirus (HCMV) infection appears to be independent from DDR induction and is directly promoted by viral proteins [27]. In cancer cells, genotoxic stress increases the sensitivity of tumor cells to NK cell-mediated lysis by the induction of NKG2DL expression mainly through DDR activation [19,20,21]. Moreover, this pathway is up-regulated by genotoxic chemotherapeutic drugs that enhance NKG2DL expression preferentially in cells undergoing senescence, thus supporting the notion that drug-induced senescence represents a mechanism that contribute to the NK cell-mediated tumor cell clearance [20,21,22].

In addition, the heat shock pathway, the oxidative stress pathway and the endoplasmic reticulum stress response may act together with the DDR pathway to ensure NKG2DL expression in unhealthy cells [3,4]. Anti-cancer therapeutic interventions may also improve the efficacy of NK cell immune-surveillance affecting NKG2DL expression in tumor cells, as demonstrated by several evidences including those obtained on the hematological cancer Multiple Myeloma (MM) [28,29,30,31,32].

Although stressing conditions ensure NKG2DL expression on transformed and infected cells, both viruses (e.g., HCMV, HIV-1) and cancer cells have developed the capability to inhibit cell-surface NKG2DL expression in order to counteract NKG2D-mediated recognition. Some viruses encoded proteins, such as UL16 and UL142 of HCMV [33,34,35] or HIV-Nef [36] can cause NKG2DL intracellular retention. During tumor progression, immunoediting processes favor the selection of tumor variants that dampen NKG2DL membrane expression producing soluble ligands through alternative splicing [37,38], proteolytic shedding mediated by metalloproteinases of A Disintegrin And Metalloproteinase (ADAM) family [39,40,41,42] or exosome secretion [43,44].

## 2. Ligand-Dependent NKG2D Down-Modulation: Impact on NK Cell Function

Although expression of NKG2DLs represents a danger signal sufficient to alert NK cells against damaged cells, in condition of chronic exposure to NKG2DL-expressing cells or soluble NKG2DLs, receptor down-modulation occurs with the consequent impairment of NKG2D-mediated cytotoxic cell functions [39,45,46,47,48,49,50,51,52].

The first evidence of NKG2D down-regulation comes from studies of Groh and co-workers on CD8^+^ tumor infiltrating T lymphocytes: NKG2D down-modulation was induced either upon chronic stimulation with target cells expressing the ligand MIC or with patients’ sera containing soluble MICA/B [45]. These findings were followed by in vitro and in vivo evidences demonstrating that engagement with both MIC and ULBP ligands induces NKG2D down-modulation on human NK cells [39,46,49,50,51]. The presence of MIC ligands in sera derived from patients with colorectal cancer correlated with a reduction of NKG2D expression on circulating NK cells that resulted unable to kill MIC^+^ autologous tumor cells [46]. NKG2D down-modulation was also observed on NK cells incubated with ULBP-transfected cells or primary ULBP2^+^ leukemic cells [39]. However, in this experimental setting NKG2D expression was not affected by soluble form of ULBP2 released in the culture supernatants by metalloproteinase-mediated shedding.

In order to analyse whether different membrane-bound NKG2DLs are equally able to induce NKG2D down-modulation, transfectants expressing MICA or ULBP2 have been co-cultured with human primary NK cells [51]. MICA and ULBP2 vary in their potential to induce NKG2D down-modulation, with MICA being the most potent ligand leading to a severe impairment of NKG2D-dependent NK cell cytotoxicity. These results suggest that the intrinsic ability of distinct ligands to reduce NKG2D expression may be due to differences in their affinity/avidity for NKG2D and/or to their mode of membrane anchor (transmembrane and GPI-linked, respectively).

With regard to the ability of human soluble ligands to down-regulate their receptor and impair NKG2D-mediated cytolytic functions, contrasting results exist. Down-modulation of NKG2D expression on CD8^+^ T cells was observed in patients with different epithelial cancers and correlated with the presence of soluble MICA/B in their sera [45]. NKG2D expression was also found reduced on circulating NK cells from patients with colorectal cancer that display the presence of soluble MIC ligand in sera [46]. In contrast, in MM patients only membrane-bound NKG2DLs induce NKG2D down-modulation, despite the presence of soluble ligands in patients’ sera [53]. Similarly, high concentrations of soluble MICA in sera of patients affected by autoimmune diseases are not associated with any changes in NKG2D surface expression [54,55,56]. In line with these data, NKG2DLs released into culture supernatants not always leads to receptor down-modulation [39,57,58]. NKG2D surface expression does not change upon stimulation with metalloproteinase-shed ULBP2 [39], while it was found reduced upon incubation with high concentrations of recombinant soluble ULPB2 [57]. On the other hand, MICB released in vitro from activated CD4^+^ lymphocyte down-regulate NKG2D expression on CD8^+^ T cells [58].

These conflicting results may be explained by the different nature of the soluble ligands (shed by proteolytic cleavage or released in exosomes). Comparing the results obtained with shed and exosome-release ligands it appears that these latter forms are more potent down-modulators than the shed counterparts. Indeed, culture supernatants containing exosome-released MICA*008 are more potent down-modulators than metalloproteinase-shed MICA [43]. Moreover, exosome-released ULBP3 decreases receptor expression more efficiently than the metalloproteinase-shed ULBP2 ligand [44]. Similar results were obtained upon chronic exposure to tumor derived MIC-expressing exosomes: they induce down-modulation of NKG2D surface expression on NK and CD8^+^ T lymphocytes, thus reducing their ability to produce IFN-γ and to kill tumor cells [50]. Moreover, during pregnancy exosomes bearing NKG2DLs are secreted by syncytiotrophoblast and contribute to immune tolerance of the fetus by reducing NKG2D surface expression and function on maternal circulating PBMCs [59]. These results may be explained considering that the surface of exosomes exposes multiple ligand molecules that engage NKG2D with high avidity.

Several lines of evidence obtained on mouse models also support a role for both human and mouse membrane-bound ligands in NKG2D down-modulation and functional impairment (Table 1). NKG2D down-modulation on activated NK cells in nonobese diabetic (NOD) mice is responsible for a reduced cytotoxicity and cytokine production [47]. In transgenic mice overexpressing MICA, NK and CD8^+^ T cells were unable to reject MICA-positive tumors due to a reduced receptor expression [49]. In addition, two transgenic models of the mouse ligand Rae-1 expression have also been used to demonstrate that NKG2D down-modulation leads to NK cell functional impairment [48,60].

In in vitro experiments, H60 membrane-bound ligand resulted in down-regulation of NKG2D expression and its associated functions in murine NK cells [61]. Regarding the soluble forms of murine NKG2DLs, it is interesting to point out that soluble MULT1 promotes tumor rejection by blocking the interaction with NKG2DLs expressed on non-tumor host cell, thus preventing continuous NKG2D down-modulation and NK cell desensitization [62]. These results strongly argued against a role of soluble monomeric murine NKG2DLs in immune evasion.

All together these evidences support the conclusion that a persistent engagement with membrane-bound or exosomal NKG2DLs down-modulates NKG2D on cytotoxic lymphocytes and ultimately, affects their killing capability towards NKG2DL-bearing target cells.

A still open question is whether NKG2D down-regulation impacts on other unrelated NK cell-activating receptor-dependent functions. Upon an overnight co-culture with MICA- and ULBP2-expressing target cells, human NK cells reduce NKG2D expression and function, while maintain intact cytotoxic function triggered by other activating receptors [51]. However, longer in vitro stimulation with NKG2DLs has been shown to impact on NKG2D unrelated functions both in human and mice. In fact, CD3ζ chain degradation was observed in human CD8^+^ T lymphocytes and NK cells upon 3 days of co-culture with MICA-transfectants [63], and in murine NK cells stimulated for 3 days with H60-expressing targets [64], thus impairing the functional capacity of CD3ζ-associated receptors.

Notably, NKG2D expression may also be regulated by ligand-independent signals. Indeed, it was extensively reported that TGF-β down-regulates human NKG2D surface expression interfering with DAP10 transcription [65,66,67]. Moreover, several cytokines including IL-2, IL-7, IL-12, and IL-15, increase NKG2D/DAP10 expression acting at transcriptional and post-transcriptional level [5,66].

Altogether these results suggest that the functional consequences of NKG2D down-modulation may depend not only on the nature of the ligand and the lengths of receptor stimulation but also on the concurrent action of cytokines.

## 3. NKG2D Endocytosis: Intracellular Receptor Trafficking and Signalling

Regarding the mechanisms of NKG2D down-modulation, several lines of evidence support a pivotal role for ligand-induced receptor endocytosis. Upon ligand binding, both murine and human NKG2D receptors are mainly internalized by clathrin-dependent endocytosis [47,58], and rapidly traffics through endosomal compartment towards lysosomes where both NKG2D and DAP10 are degraded [45,51,68]. However, receptor engagement with MICA is more rapidly followed by lysosomal degradation if compared to ULBP2 [51], suggesting that the rate of receptor internalization and degradation depends on the nature of the ligand. These differential effects may be explained by a selective activation of the ubiquitin pathway that promotes a more rapid receptor endocytosis. Indeed, the ubiquitin ligase c-Cbl is required for MICA- but not ULBP2-induced NKG2D endocytosis [51]. Notably, in human NK cells NKG2D internalization and degradation depend on DAP10 ubiquitination [69]. In line with these results, in a murine model NKG2D/DAP10 degradation was observed when DAP10 was fused with a molecule of ubiquitin [70], suggesting that this pathway can be also implicated in murine NKG2D endocytosis.

Regarding the fate of murine internalized receptors, conflicting results exist. The expression level of both NKG2D associated DAP10 and DAP12 was reduced after stimulation of murine NK cells with transfectants over-expressing the NKG2DL H60 [61], whereas NKG2D internalization was not followed by receptor degradation upon interaction of murine NK cells with Rae1ε-bearing targets [47]. These findings suggest that also in mouse the kind of NKG2DLs and/or the length of NKG2D/NKG2DL stimulation differently impacts on the fate of internalized receptor complexes.

Even though persistent stimulation with cognate ligands promotes receptor down-modulation and dampens NK cell functions, recent evidences demonstrated that ubiquitin-dependent NKG2D endocytosis is indispensable for the propagation of intracellular signals leading to human NK cell function [52,69]. Indeed, although proximal events occur at the plasma membrane, the full activation of Extracellular signal-Regulated Kinases 1 and 2 (ERK1/2) requires receptor internalization and sorting in endosomal compartments [69]. NK cells in which NKG2D internalization is inhibited resulted in impairment of both their cytotoxic function and cytokine production [69]. These results demonstrate that NKG2D continues to signal upon internalization, but also support the notion that endocytosis elicits a fundamental role in the regulation of the NKG2D-dependent functional program.

**Figure 1:**
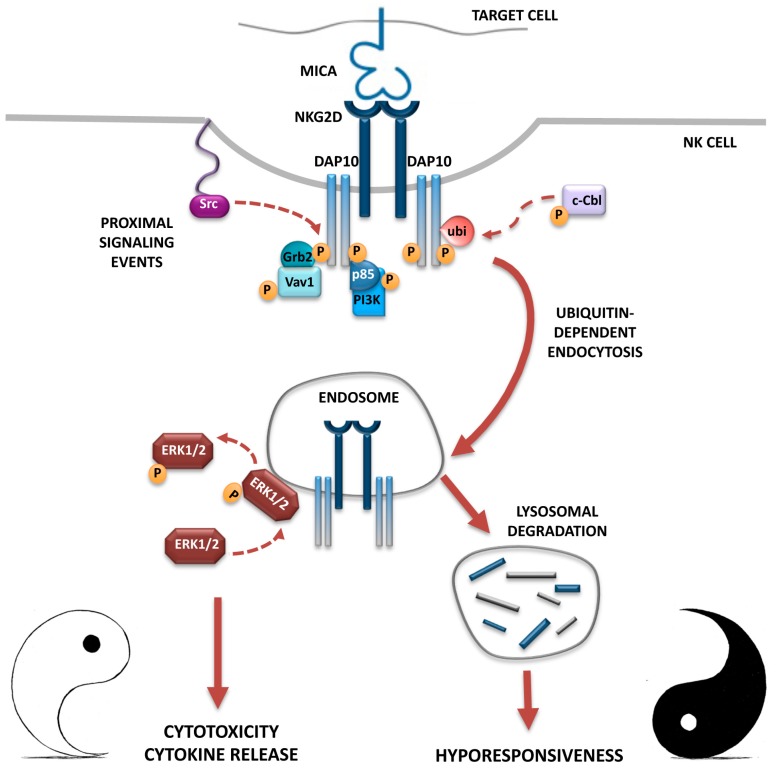
The Yin and Yang of NKG2D endocytosis: functional consequences on NKG2D-mediated signaling and NK cell effector functions. In human NK cells, NKG2D engagement by MICA induces Vav1 phosphorylation and PI3K activation, which are responsible for the initiation of intracellular signals leading to effector functions. On the other hand, MICA engagement activates the ubiquitin ligase c-Cbl and promotes DAP10 ubiquitination that provides a signal responsible for NKG2D internalization and lysosomal degradation, that ultimately induces a hyporesponsiveness towards NKG2DL-bearing target cells (the black part of the Yin-Yang symbol). However, internalized NKG2D receptors before being degraded continue to signal from endosomes: they promote ERK1/2 phosphorylation allowing the full activation of NK cells (the white part of the Yin-Yang symbol)*.* NKG2D/DAP10 receptor complexes are depicted with intact rectangles (cell surface membrane and endosomes), and with fragmented rectangles (lysosomes) to indicate that their degradation was occurred. Arrows represent relationships that were well established (solid lines) or not yet demonstrated (dashed lines). Modified from Quatrini et al. [69].

Altogether these data support a model in which the internalized NKG2D/DAP10 complexes contribute to the recruitment of ERK, which is a critical signal element in driving NK cell cytotoxicity [71], to endosomal compartments where ERK is phosphorylated and activated, thus allowing signal propagation and functional responses. On the other hand, persistent stimulation and receptor endocytosis leads to lysosomal degradation of activated receptor complexes and dampens NKG2D-mediated functions (Figure 1). Whether murine NKG2D that mediates ITAM-dependent signals may also require receptor endocytosis is currently unknown.

Endosomes can function as platforms to initiate and/or to sustain receptor-mediated signals, as supported by several findings that document a close relationship between endocytosis and signalling.

In the context of ligand-induced down-regulation of receptor tyrosine kinases (RTKs) as well as G protein-coupled receptors (GPCR) [72,73], the rate of ligand-induced receptor internalization is very high with respect to the rate of receptor degradation, and this long receptor residence in endosomes serves to sustain the signalling.

Several evidences support the concept that endosomes can act to initiate and/or to sustain receptor-mediated signal also in immune cells. The Toll-like Receptors (TLR) TLR3, TLR7, and TLR9 initiate signalling upon their ligand-induced internalization [74], whereas TLR4 activates different signalling pathways depending on its cellular location, regulating the production of diverse inflammatory cytokines [75]. The role of endosomes has also been demonstrated for B and T cell receptors-mediated signalling. In those cases, internalized receptors ensure the appropriate extent and strength of signalling, respectively [76,77]. Regarding NK cells, the activating receptor KIR2DL4 accumulates into early endosomes in order to initiate a pro-inflammatory cascade [78,79]. With respect to the NKG2D-DAP10 complex on human NK cells, the finding that internalized receptors are rapidly degraded [69], suggests that endosomal signalling is required to amplify MAPK/ERK signal but not to sustain it.

In conclusion, these results provide new insight on the role of the endosome in NKG2D-mediated signal propagation and regulation of NK cell functions that could be extended to other NK cell activating receptors.

## 4. Down-Modulation of Other Activating NK Cell Receptors and Their Impact of NK Cell Function

Besides NKG2D, NCRs, DNAM1 and CD16 are the best-characterized activating NK cell receptors implicated in immune responses against cancer [1]. Interestingly, numerous evidences have revealed alterations of the surface expression of those NK cell receptors upon sustained engagement with their respective ligands in tumor-patients [80,81,82,83,84,85,86,87,88].

NCRs comprise NKp44, NKp30, and NKp46 [89], and all of them have been implicated in anti-tumor immune responses on the basis of the ability of monoclonal antibodies (mAbs) against these receptors to block human NK cell killing of various tumor cell lines [90]. In many cases, combining the Abs against NKp30, NKp44 and NKp46 resulted in more efficient blocking of NK-mediated tumor cell lysis than the same Abs used individually, suggesting the existence of multiple ligands on the target cells*.* However, the full identification of NCR ligands remains to be performed. The only cell surface ligand known to bind to an NCR is the NKp30 ligand B7-H6, a member of the B7 family exclusively expressed on tumor cells [91]. The importance of this receptor family in the context of NK cell-mediated tumor immune-surveillance raises the possibility that cancer cells can shape NCR expression in order to prevent NK cell recognition. Indeed, upon direct contact with leukemic cells a reduced NKp30 and NKp46 expression was observed on NK cells derived from acute myeloid leukemia (AML) patients [80]. In line with these results, reduced NKp30 level was observed on NK cells derived from peritoneal fluid of ovarian carcinoma patients compared to autologous peripheral blood NK cells [85]. NKp30 down-modulation is a consequence of chronic stimulation with both tumor cell expressing the NKp30 ligand B7-H6 and soluble B7-H6 present in peritoneal fluid. Consequently, NK cells showing an NKp30^low^ phenotype resulted impaired in both cytotoxic function and IFNγ production when stimulated with B7-H6 bearing target cells [85]. Similarly, high levels of soluble B7-H6 ligand in the sera of neuroblastoma patients correlates with NKp30 down-modulation on circulating NK cells and impaired NKp30-dependent NK cell activation and disease progression [86]. All together these results suggest that, in the case of B7-H6 ligand, both membrane-bound and soluble molecule are equally able to reduce surface receptor expression.

DNAM1 receptor binds to the poliovirus receptor CD155 and the Nectin adhesion molecule CD112, both up-regulated in stressed cells, and has a pivotal role in preventing spontaneous tumor formation and in controlling tumor growth [92]. However, evidence exist that chronic stimulation with DNAM1 ligands in the context of tumor transformation provokes receptor down-modulation on NK cells [81,82,83]. Significant DNAM1 down-modulation was observed on NK cells derived from MM patients compared to healthy donors [81], although a role of MM-expressed DNAM1 ligands has not been directly assessed. In line with these results, NK cells from peritoneal fluids of ovarian carcinoma patients show reduced DNAM1 surface expression levels as a consequence of persistent stimulation with CD155 expressed on cancer cells [82]. Notably, DNAM1 down-modulation required cell-cell contacts, thus excluding the contribution of soluble CD155 present in peritoneal fluid [82]. Moreover, in NK cells from AML patients, down-modulation of DNAM1 receptor is induced by a direct contact with leukemic blasts expressing both CD155 and CD112 DNAM1 ligands, and leads to an impaired natural cytotoxicity [83]. Altogether these results support a role for cell membrane DNAM1 ligands, but not for their soluble counterpart, in promoting receptor down-regulation.

The molecular mechanisms responsible for DNAM1 and NCR down-modulation have not been clarified yet. In particular, it would be interesting to investigate the involvement of either endocytosis or metalloproteinase-mediated shedding and intracellular transfer during effector-target interaction (trogocytosis).

CD16 (FcγRIIIA) is a potent activating receptor expressed on the CD56^dim^CD16^+^ NK cell subset that mediates ADCC and cytokine release [93] through the tyrosine phosphorylation of its transducing subunits [94,95]. In particular, ADCC activity has been associated with better outcomes for some type of cancers [96]. Many therapeutic monoclonal antibodies (mAb) that specifically recognize tumor cells are able to bind to CD16 on NK cells, promoting NK cell-mediated ADCC of these tumor cells [97,98]. In response to mAb-coated tumor cells, however, CD16 is down-modulated from the cell surface of NK cells, leading to impaired NK cell function and decreasing the efficacy of Ab-based therapies [84,87,88]. Thus, identification of the molecular mechanism(s) responsible for CD16 down-regulation has clinical significance. Several findings have reported that Ab-mediated CD16 down-modulation is mainly a consequence of metalloproteinases (MMPs)-induced shedding [99,100,101,102]. Indeed, treatment with MMPs inhibitors preserved CD16 expression and restored impaired NK cell-mediated ADCC [84]. Notably, CD16 down-regulation also depends on internalization of cross-linked receptors as reported upon anti-CD16 mAb engagement [103,104] or upon co-culture with tumor target cells opsonized with therapeutic Abs [84,87,88]. Receptor endocytosis requires an integral and functional cytoskeleton [104], a clathrin-dependent pathway (Molfetta, preliminary results), and is followed by ubiquitin-dependent degradation of CD16-associated signaling elements [88,105,106]. Intriguingly, as a result of this NK cells are not only impaired in their ability to further perform ADCC, but also to induce spontaneous cytotoxic activity triggered by unrelated activating receptors including NKG2D, DNAM1 and NCRs, while they maintain the ability to produce IFNγ [88]. It would be interesting to investigate in the future whether CD16 endocytosis is required to activate the functional program of NK cells, as formally demonstrated in the case of NKG2D.

## 5. Concluding Remarks

NKG2D is one of the main NK cell activating receptor involved in anti-tumor and anti-viral immune response. However, persistent exposure to NKG2D ligand-expressing target cells promotes NKG2D down-modulation that leads to lysosomal receptor degradation with the consequent impairment of NKG2D-mediated functions. Intriguingly, receptor complexes before being degraded promote ERK1/2 phosphorylation from endosomes and ensure NK cell full activation.

Ligand-induced down-modulation from the cell membrane has been demonstrated also for NK cell activating receptors other than NKG2D. It would be interesting to investigate whether these receptors are down-modulated by endocytosis, and whether they can signal from endosomal compartments. Considering that NK cell activation requires the co-engagement of at least two activating receptors, endosomal membranes would provide a platform for the integration of these signals, thus ensuring a better control of NK cell response.

## Figures and Tables

**Table 1:** In vivo models demonstrating that chronic exposure to NKG2DLs results in receptor down-regulation.

Experimental Mouse Model	Findings	Reference
Nonobese diabetic (NOD) mice	NKG2D down-modulation on activate NK cells Impaired NKG2D-dependent NK cell functions	[47]
FVB transgenic mice overexpressing Rae-1ε ligand ubiquitously or localized in normal epithelium	NKG2D down-modulation on splenic NK cells (and intraepithelial T cells) Impaired NKG2D-dependent NK cell functions Normal generation of antigen (HY) specific CTL memory Increased susceptibility to tumorigenesis	[48]
C57BL/6 transgenic mice constitutively and ubiquitously overexpressing MICA	NKG2D down-modulation on splenic NK cells Impaired NKG2D-dependent functions in vivo and in vitro Impaired CD8 T cell response to L. monocytogeneses	[49]
C57BL/6 transgenic mice ubiquitously overexpressing Rae-1ε ligand	NKG2D down-modulation on splenic NK cells Impaired NKG2D-dependent NK cell functions Normal NK and CD8 T cell response to MCMV	[60]

NKG2D, natural-killer receptor group 2, member D; NK, natural killer; CTL, cytotoxic T lymphocytes; MCMV, murine cytomegalovirus.
